# The accuracy of VO_2_ max, heart rate and energy expenditure measurements from Google Pixel Watch 3

**DOI:** 10.1371/journal.pone.0356808

**Published:** 2026-09-15

**Authors:** Rory Lambe, Moritz Schumann, Sherene Hamilton, Lauren Donnelly, Thomas White, Seán O’Reilly, Saoirse Lally, Cailbhe Doherty

**Affiliations:** 1 School of Public Health, Physiotherapy and Sports Science, University College Dublin, Dublin, Ireland; 2 Rinn Artificial Intelligence, University College Dublin, Dublin, Ireland; 3 School of Medicine and Health, Experimental Exercise Science, Technical University of Munich, Munich, Germany; Polytechnic University of Marche: Universita Politecnica delle Marche, ITALY

## Abstract

Consumer wearables enable scalable physiological assessment and pre-symptomatic disease detection. However, validation of wearable data remains insufficient in scope and rigour, hindering integration into clinical and public health practice. Here, we evaluated the validity of VO_2_max, heart rate and energy expenditure from the Google Pixel Watch 3. Thirty-four participants completed a maximal exercise treadmill test using indirect calorimetry, while wearing a Polar H10 chest strap. Pixel Watch estimates of VO_2_max were generated in free-living conditions; heart rate and energy expenditure estimates were recorded during exercise testing. Bland-Altman analysis showed low bias but wide limits of agreement for VO_2_max estimates from the Pixel Watch 3 (bias −0.53 mL/kg/min; limits of agreement [LoA] −12.56 to 11.50; mean absolute percentage error [MAPE] 8.65%). Heart rate demonstrated strong agreement with the criterion overall, despite measurement variability (bias −2.39 bpm; LoA −24.42 to 19.65; MAPE 2.73%). The error for energy expenditure varied substantially between participants (bias −8.43 kcal; LoA −72.75 to 55.88; MAPE 15.02%). These results indicate that VO_2_max estimates lack sufficient individual-level validity for clinical use, although the bias suggests utility for population health monitoring. Heart rate showed acceptable error for exercise monitoring, whereas the large variation in error for energy expenditure limits its practical use. Our findings illustrate that wearable data require condition- and population-specific validation before incorporation into health care pathways.

## Introduction

Over 500 million people worldwide own a consumer wearable that continuously monitors their health, offering a substantial opportunity to improve proactive disease management [1]. Wearables measure numerous health metrics predictive of illness and mortality, including vital signs and circadian rhythms [2,3], and they have already been used to monitor population-level physical activity, to predict metabolic dysfunction, and to detect disease before symptom onset [4–7].

Despite their capability and ubiquity, wearables remain sparsely integrated in public health and clinical care, driven by a lack of measurement validation. Less than 5% of metrics across all consumer wearables have been validated to date, with only 11% of devices validated for at least one metric [8]. Without robust validation, users are unaware whether wearable device outputs accurately reflect their physiology, researchers cannot use wearable data as digital endpoints in clinical trials, and clinicians lack sufficient evidence to incorporate measurements into care.

This validation deficit is particularly consequential for metrics with direct clinical relevance. Cardiorespiratory fitness, quantified by maximal oxygen uptake (VO_2_ max), has a strong inverse association with all-cause mortality and has been proposed as a clinical vital sign by the American Heart Association [9]. In addition, vigorous physical activity – classified using heart rate – and energy expenditure are each associated with cardiovascular and all-cause mortality [10,11].

Validation efforts to date show that accuracy varies substantially between these metrics, influenced by device and measurement conditions [12]. Substantial error has been reported for VO_2_ max and energy expenditure [13–15], whereas heart rate has shown stronger agreement with criterion measures [16]. Yet the existing literature lacks rigour and adherence to best-practice validation guidelines: Studies frequently rely on inappropriate statistical methods and ad-hoc protocols [17]. Moreover, much of the literature evaluates hardware and software that have since been discontinued. To our knowledge, there has been no validation of the most recent models from Apple, Google or Garmin.

Fitbit devices have been the most widely used consumer wearables in research to date, but with Google’s acquisition of Fitbit in 2021, the Google Pixel Watch has become the flagship model in the Google-Fitbit ecosystem. Despite this, there has been little independent validation of its measurements, and although the device incorporates Fitbit’s algorithms, accuracy cannot be inferred from previous validation of Fitbit models given the evolution of sensors and software. Here, we assessed the analytical validity of VO_2_ max, heart rate and energy expenditure estimates from the Google Pixel Watch 3 to inform their use in individual health monitoring, research and public health practice.

## Methods

### Ethics statement

Ethical approval was granted by the University College Dublin Human Research Ethics Committee (LS-23–66) on February 7, 2024. Written informed consent was obtained from participants.

### Study design and eligibility criteria

This cross-sectional validation study was conducted in Dublin, Ireland between February 13, 2025 and August 1, 2025. We recruited a purposive convenience sample from the greater Dublin area. Healthy adults, aged 18 years or older, were recruited via posters, email and word-of-mouth. This included university staff and students, recreational athletes and individuals who were physically inactive. We excluded pregnant women, individuals with a contraindication to maximal intensity exercise and those using medication affecting cardiovascular function. All participants completed the Physical Activity Readiness Questionnaire Plus (PAR-Q+) prior to enrolment. The study protocol was designed and reported in accordance with the six domains outlined by the Towards Intelligent Health and Well-Being Network of Physical Activity Assessment (INTERLIVE) consortium in their best-practice recommendations for validation of consumer wearables [17]. The Pixel Watch 3 was evaluated according to the *analytical validation* component of the V3 framework for evaluating biometric monitoring technologies [18].

### Testing protocol

Each participant completed a maximal cardiopulmonary exercise test (CPET) using indirect calorimetry at the Institute for Sport and Health, University College Dublin. Indirect calorimetry is the gold standard for VO_2_ max assessment and is a validated criterion measure for energy expenditure [15,19]. An exercise treadmill test was conducted according to the American College of Sports Medicine’s (ACSM) Guidelines for Exercise Testing and Prescription, following the modified Åstrand protocol [19,20]. A treadmill speed between 8 and 13 km/h was selected, which remained constant throughout. Following an initial three-minute warm-up period at 0% incline, the treadmill incline was increased by 2.5% every two minutes. Before exercise testing, height, body mass and skin type were recorded. Skin type was assessed using the Fitzpatrick Skin Phototype Scale, which categorises human skin into six types based on melanin content and reaction to sun exposure [21]. Exercise testing was conducted on the h/p/cosmos Venus treadmill (h/p/cosmos, Nußdorf-Traunstein, Germany). Participants were instructed to refrain from caffeine and nicotine for 12 hours prior to testing, and to avoid strenuous activity and alcohol for a minimum of 24 hours [17].

To ensure true VO_2_ max had been attained, participants were required to meet at least two of the following criteria: heart rate within ±10 bpm of age-predicted maximum (220 – age); respiratory exchange ratio (RER) ≥ 1.15; rate of perceived exertion (RPE) ≥ 17; VO_2_ plateau, defined as an increase in VO_2_ of less than 150 mL/min, with an increase in work rate as evidenced by physiological data [19]. If two or more criteria were not met, the value was regarded as a VO_2_ peak and excluded from VO_2_ max analyses. Each participant’s cardiorespiratory fitness level, determined by indirect calorimetry, was classified in accordance with the reference standards of the Fitness Registry and the Importance of Exercise National Database (FRIEND) [22].

### Criterion methods

Indirect calorimetry measurement of VO_2_ max and energy expenditure was conducted using the COSMED Quark CPET metabolic cart (COSMED, Trentino, Italy), which was calibrated prior to each participant’s test according to manufacturer instructions. This included gas and flow metre calibration. An open-circuit breath-by-breath method was used. Heart rate was measured using the Polar H10 (Polar Electro Oy, Kempele, Finland), a validated electrocardiography chest strap, which participants wore in accordance with manufacturer guidelines [23,24]. A ‘Treadmill Running’ workout was recorded using the Polar Beat mobile application for the duration of exercise testing.

### Index measure: Google Pixel Watch 3

Participants wore a Pixel Watch 3 during exercise testing. The device was placed snugly on each participant’s wrist, proximal to the ulnar styloid process, as per manufacturer guidelines. A ‘Treadmill Run’ was recorded to measure heart rate and energy expenditure at the highest available measurement frequency. Prior to testing, the research team inputted the participant’s demographic data in their Fitbit mobile application. This included height, body mass, date of birth and sex.

Following exercise testing, participants were provided with the Pixel Watch 3 to generate a VO_2_ max estimate. They were asked to wear the device as consistently as possible, during day and night, and were informed of manufacturer guidelines for devices wear. The Pixel Watch 3 estimates VO_2_ max based on users’ heart rate response during outdoor running, combined with GPS and demographic data (age, sex, height, body mass). In accordance with this procedure, participants were requested to independently record several outdoor runs of 15 minutes or longer in duration on flat terrain, with GPS enabled, until a VO_2_ max estimate had been generated. Estimates were required to be generated within one week of criterion testing. All Pixel Watch 3 devices were running Wear OS 5.1.

### Data processing

For analysis of VO_2_ max, breath-by-breath data from the COSMED Quark CPET were filtered using a 30-second moving time average and exported to Microsoft Excel. The highest time-averaged VO_2_/kg value was interpreted as VO_2_ max. This was compared to the VO_2_ max estimate from the Pixel Watch 3 that was obtained on the date closest to criterion testing.

To analyse energy expenditure, the raw data from the COSMED Quark CPET were exported and time-matched to the start and end of the Pixel Watch 3 ‘Treadmill Run’. The total energy expenditure value from the COSMED device, computed using the Weir equation [25], was compared with the ‘Energy burned’ value in the Fitbit app, which represented the total energy expenditure estimate from the Pixel Watch 3 for the exercise testing session.

Heart rate data from the Polar H10, recorded at a sampling frequency of one measurement per second, were downloaded through the Polar Flow web application. Artefacts in Polar H10 heart rate data – defined as gaps in recording, or deviation in adjacent heart rate values of ±10 bpm or more with subsequent normalisation – were removed prior to analysis. The Pixel Watch 3 heart rate data were downloaded via Fitabase (Small Steps Labs, California). The sampling frequency of the Pixel Watch 3 was irregular, with readings occurring every one to four seconds. Only heart rate measurements with matching timestamps were compared; Polar H10 measurements without corresponding Pixel Watch 3 measurements were not included in analyses. There was no post-processing of Pixel Watch 3 heart rate data.

### Outcomes

Our primary outcome was the agreement of VO_2_ max estimates from the Pixel Watch 3 with indirect calorimetry, and the secondary outcomes were the agreement of heart rate and energy expenditure estimates with the Polar H10 and indirect calorimetry, respectively.

### Statistical analysis

The target sample size was calculated based on the accuracy reported in previous validation studies of wearable devices [26]. We conducted Bland-Altman limits of agreement analysis for each outcome [27]. The mean difference (bias) was calculated as the values from the Pixel Watch 3 minus the criterion measure. For VO_2_ max and energy expenditure, the 95% limits of agreement were defined as the mean difference ±1.96 times the standard deviation of the differences. We calculated 95% confidence intervals for the limits of agreement, in line with methods described by Bland and Altman [28].

As a time-series of heart rate measurements was recorded for each participant, we used a mixed-effects model to calculate limits of agreement, which accounts for the dependence of observations and repeated-measures, as outlined by Bland and Altman [29]. We summed within- and between-participant variances to calculate total variance [29]. The limits of agreement were then calculated as the mean difference ±1.96 times the standard deviation of the total variance [29]. Bland-Altman plots were generated to visually illustrate the agreement.

Mean absolute percentage error and mean absolute error were also calculated for each outcome. For heart rate, mean absolute percentage error and mean absolute error were first calculated by participant to account for subject-level dependence; the overall mean absolute percentage error and mean absolute error values were the mean of the participant-level mean absolute percentage error and mean absolute error values.

Analyses were conducted in Python (version 3.13) using pandas, Matplotlib, Plotly and NumPy packages. The code used is available at github.com/rorylambe/googlepixelwatch-validation.

## Results

### Participant baseline characteristics

Thirty-four participants were recruited. All participants completed criterion VO_2_ max testing and attained VO_2_ max. Eight participants (6 male, 2 female) did not generate a VO_2_ max estimate with the Pixel Watch 3 within a week of criterion testing due to low device wear-time or failure to record a sufficient number of GPS-enabled runs. Polar H10 heart rate data was lost for one participant due to a synching failure, and energy expenditure estimates from the Pixel Watch 3 were missing for four participants. Therefore, 26, 33 and 30 participants were included in the analyses of VO_2_ max, heart rate and energy expenditure, respectively. Baseline characteristics of all recruited participants, including BMI and Fitzpatrick skin tone, are listed in Table 1. Characteristics of the participants included in analyses of VO_2_ max, heart rate and energy expenditure analyses are provided in the appendix (S1 in S1 File).

**Table 1:** Participant characteristics. Characteristics of all participants at baseline.

Characteristic	Value
Age, mean (SD), years	29.12 (8.37)
Age range, years	19–54
Female, no. (%)	12 (35%)
Height, mean (SD), cm	173.87 (9.29)
Body mass, mean (SD), kg	73.07 (10.78)
BMI, mean (SD), kg/m^2^	24.04 (1.78)
BMI range, kg/m^2^	18.98–27.19
Fitzpatrick skin tone ^a^	
Type I	3
Type II	21
Type III	8
Type IV	2
CRF level ^b^	
90^th^ percentile, no.	14
80^th^ percentile, no.	7
70^th^ percentile, no.	4
60^th^ percentile, no.	5
50^th^ percentile, no.	1
40^th^ percentile, no.	3

BMI: Body Mass Index.

^a^Number of participants of each skin tone according to the Fitzpatrick Scale.

^b^Number of participants of each cardiorespiratory fitness level, classified according to FRIEND.

### Agreement of VO_2_ max estimates with indirect calorimetry

**Fig 1:**
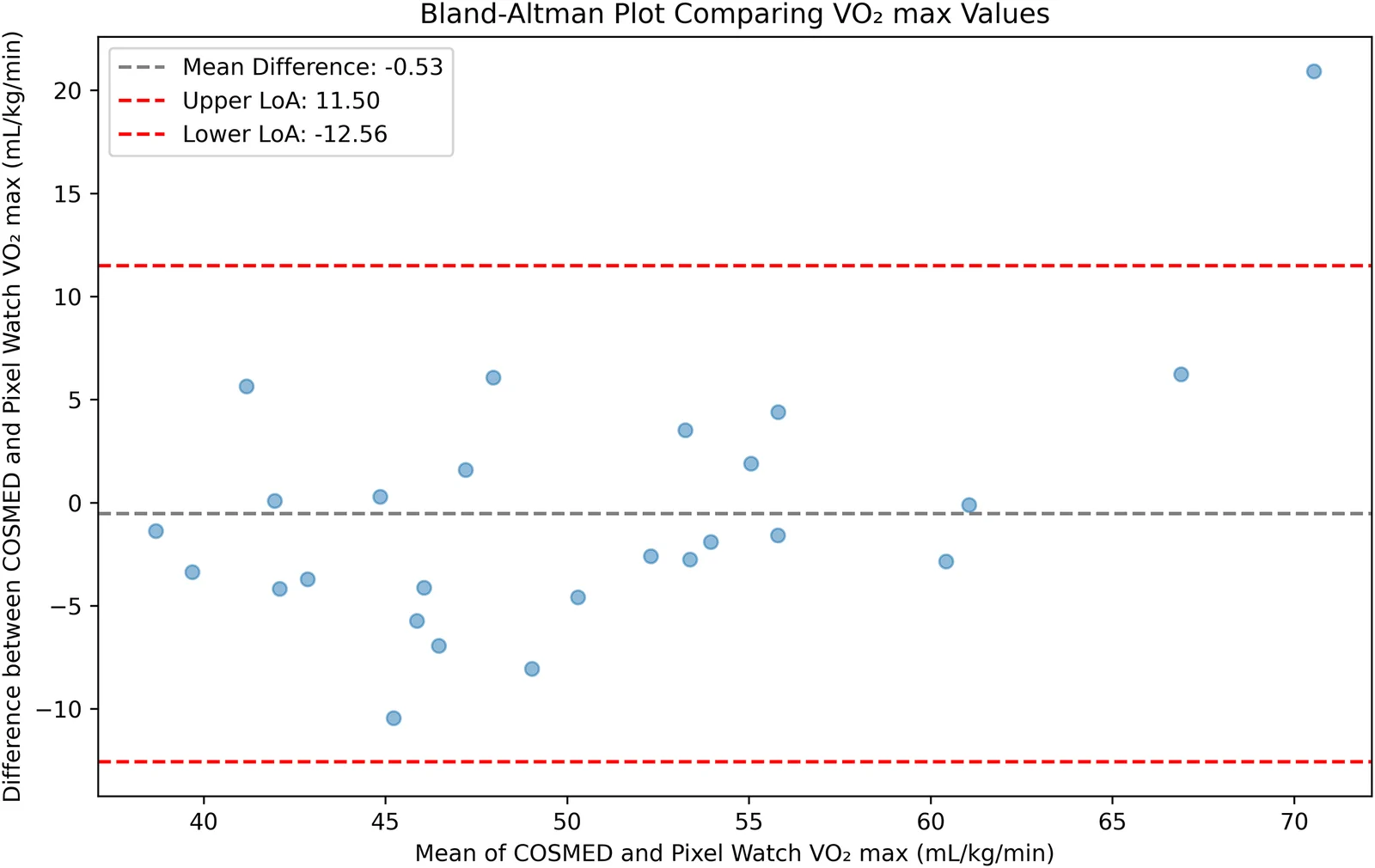
VO_2_ max Bland-Altman plot. Agreement between VO_2_ max estimates obtained from the Google Pixel Watch 3 and VO_2_ max measured using indirect calorimetry.

The Pixel Watch 3 underestimated VO_2_ max relative to the criterion, with a bias of −0.53 mL/kg/min (95% CI −3.01 to 1.95). The limits of agreement indicated measurement variability (LoA −12.56 to 11.50; Fig 1). The mean absolute percentage error was 8.65% (95% CI 5.66 to 11.63), and the mean absolute error was 4.42 mL/kg/min (95% CI 2.72 to 6.12). The Pixel Watch 3 correctly classified the FRIEND cardiorespiratory fitness percentile of eight (30%) participants and classified a further 14 (54%) within one adjacent percentile band of their actual band. Four (15%) participants were misclassified by two percentile bands, or more. A box plot illustrating the distribution of absolute error is provided in the appendix (S4 in S1 File).

### Agreement of heart rate measurements with the Polar H10

**Fig 2:**
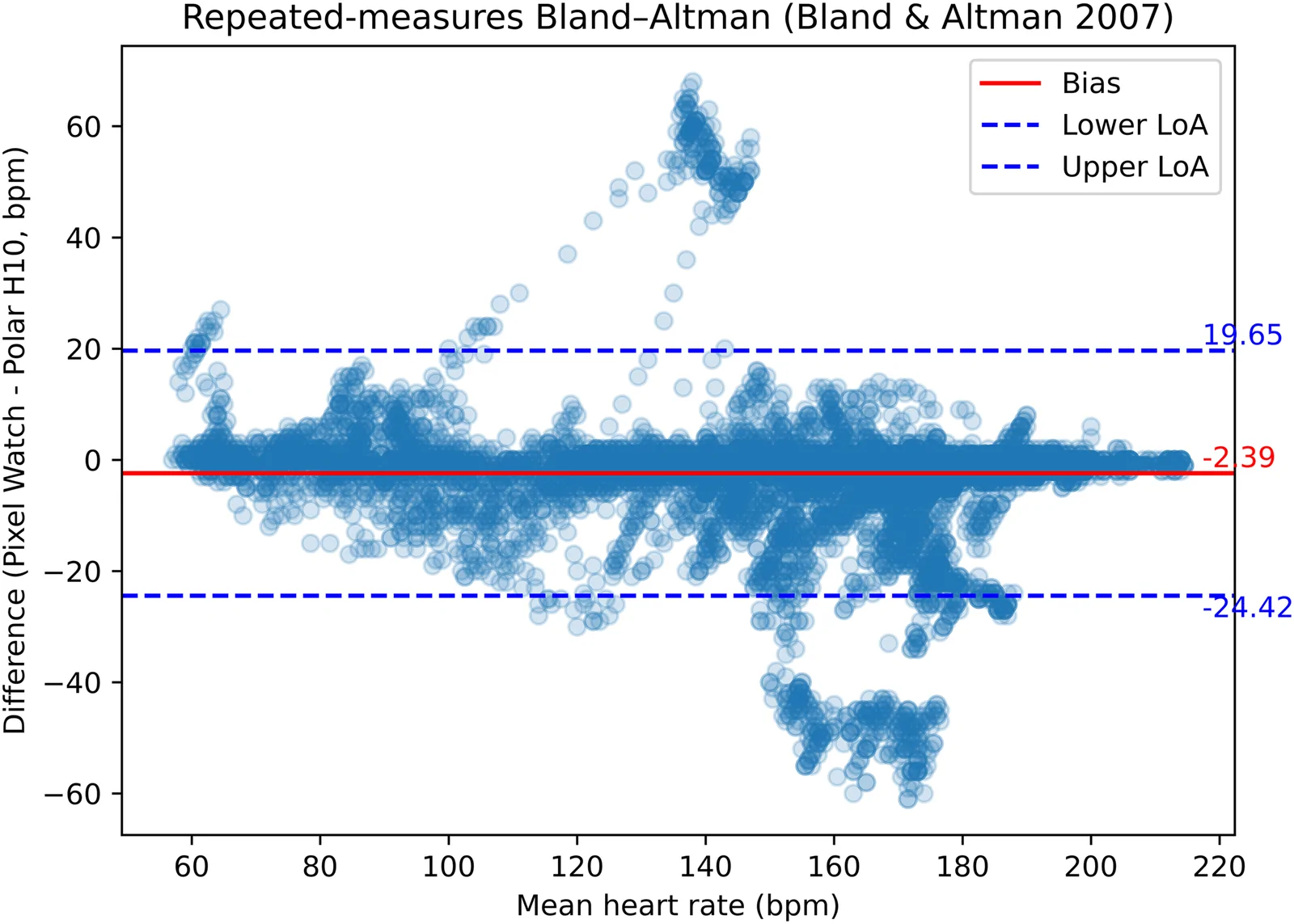
Heart rate Bland-Altman plot. Agreement between heart rate measurements obtained from the Google Pixel Watch 3 and the Polar H10.

In total, there were 11,826 paired heart rate measurements, corresponding to 45.6% (11,826/25,919) of all Polar H10 readings. The Pixel Watch 3 measured heart rate at intervals of one to four seconds. The Pixel Watch 3 underestimated heart rate, with a bias of −2.39 bpm (95% CI −5.01 to 0.16). The wide limits of agreement demonstrated substantial measurement variability (−24.42 to 19.65; Fig 2). Mean absolute error was 4.06 bpm (95% CI 1.67 to 6.45) and mean absolute percentage error was 2.73% (95% CI 1.21 to 4.25).

Upon visual inspection of the data, we identified two participants where the Pixel Watch 3 measurements deviated substantially from the criterion, by up to 61 bpm. To investigate the effect of these data on analysis, we conducted exploratory post-hoc analysis excluding these participants. Limits of agreement were substantially narrower (bias −2.01 bpm (95% CI -2.68 to -1.02), LoA −12.12 to 8.09). Example line plots of heart rate data are provided in the appendix (S2 in S1 File), with additional figures available via this study’s GitHub repository.

### Agreement of energy expenditure estimates with indirect calorimetry

**Fig 3:**
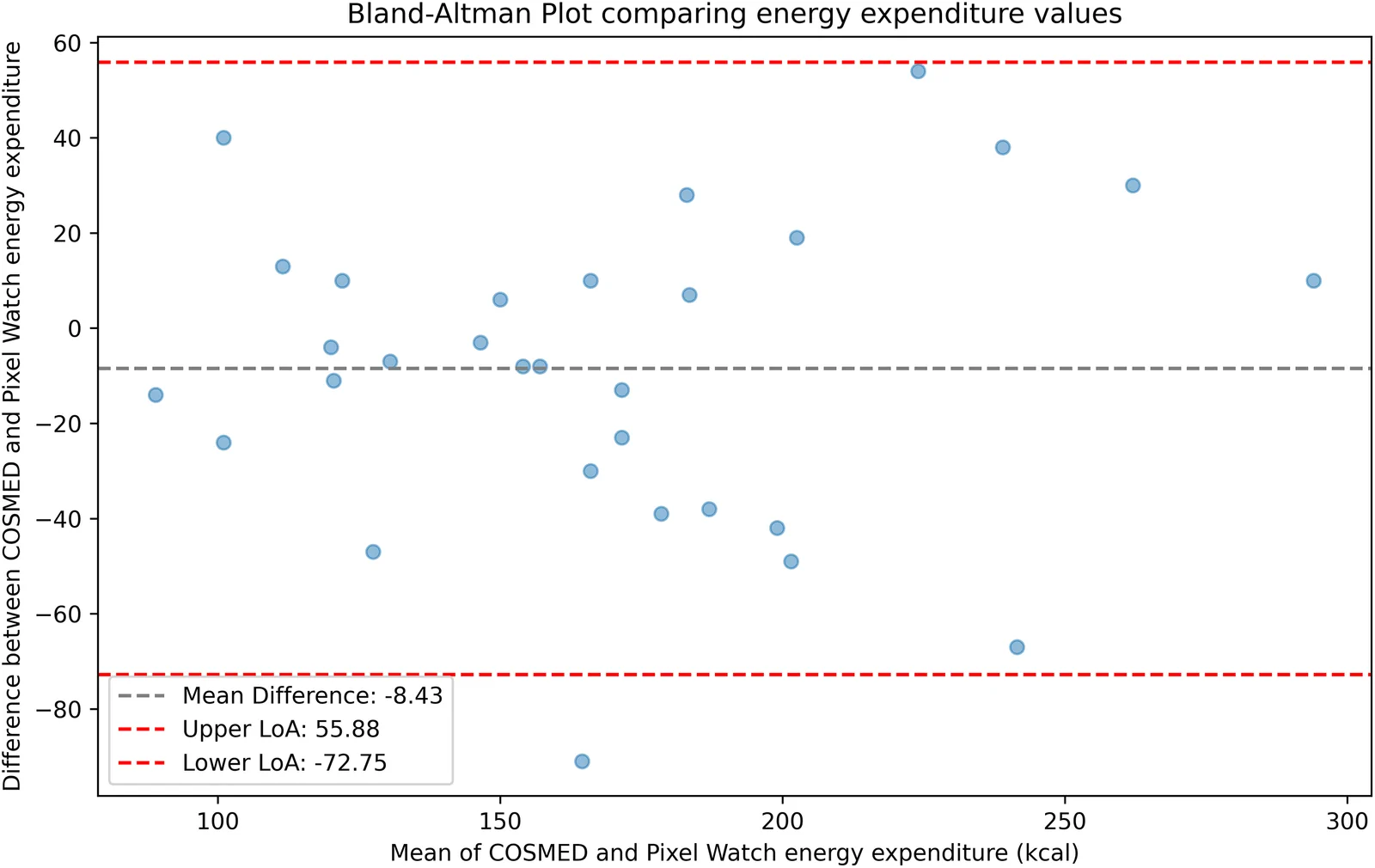
Energy expenditure Bland-Altman plot. Agreement between energy expenditure estimates obtained from the Google Pixel Watch 3 and energy expenditure measured using indirect calorimetry.

The Pixel Watch 3 underestimated energy expenditure, with a bias of −8.43 kcal (95% CI −20.69 to 3.82). The limits of agreement were wide, reflecting substantial measurement variability (LoA −72.75 to 55.88; Fig 3). The mean absolute error was 26.10 kcal (95% CI 18.22 to 33.98), and the mean absolute percentage error was 15.02% (95% CI 10.70 to 19.33). A box plot illustrating the distribution of absolute error is provided in the appendix (S3, S4 in S1 File). The results are summarised in Table 2.

**Table 2:** Summary of results. Statistical agreement between Google Pixel Watch 3 and criterion methods for VO_2_ max, heart rate and energy expenditure (EE).

Statistical measure	VO_2_ max (mL/kg/min)	Heart rate (bpm)	EE (kcal)
Mean (SD)			
Google Pixel Watch 3	50.04 (10.18)	151.68 (32.06)	164.63 (53.78)
Criterion method	50.57 (6.99)	154.06 (33.36)	173.07 (50.94)
Standard error of the mean			
Google Pixel Watch 3	2.00	2.83	9.82
Criterion method	1.37	2.88	9.30
Mean difference (95% CI)	−0.53 (−3.01 to 1.95)	−2.39 (−5.01 to 0.16)	−8.43 (−20.69 to 3.82)
Standard deviation of the differences	6.14	11.24	32.81
Bland-Altman limits of agreement			
Lower limit of agreement (95% CI)	−12.56 (−16.85 to −8.27)	−24.42	−72.75 (−93.04 to −52.46)
Upper limit of agreement (95% CI)	11.50 (7.21 to 15.80)	19.65	55.88 (35.59 to 76.18)
Mean absolute percentage error (95% CI)	8.65% (5.66 to 11.63)	2.73% (1.21 to 4.25)	15.02% (10.70 to 19.33)
Mean absolute error (95% CI)	4.42 (2.72 to 6.12)	4.06 (1.67 to 6.45)	26.10 (18.22 to 33.98)

## Discussion

In this study, we addressed the lack of validation of consumer wearables, a notable gap in the current literature given their widespread use for personal health monitoring and their growing integration into health care, where data are used for non-invasive longitudinal assessment. We assessed the validity of VO_2_ max, heart rate and energy expenditure estimates from the Google Pixel Watch 3 for use in individual health monitoring, research and public health practice. Our results showed substantial variability in error between participants for both VO_2_ max and energy expenditure. Heart rate demonstrated strong agreement with the criterion overall.

As per Bland and Altman, the limits of agreement are the key measure when determining measurement performance [29]. We found that the difference between VO_2_ max from the Pixel Watch 3 and indirect calorimetry is expected to be in the range of −12.11 to 11.66 mL/kg/min for 95% of the population, a range of approximately 24 mL/kg/min. This equates to clinically significant error, given that an increase of 3.5 mL/kg/min in VO_2_ has been associated with an 11% decrease in all-cause mortality risk [30]. The bias (−0.53 mL/kg/min) suggested that individual error may be attenuated at the population level. Notably, the limits of agreement were comparable to those reported for traditional submaximal VO_2_ max estimates, which have also shown substantial variability (~±7 mL/kg/min) [31]. The ACSM acknowledge that conventional estimation methods are clinically valuable due to their reliability, despite limited validity. Consequently, they are widely used in clinical practice to monitor patients with cardiovascular disease. This highlights the need to evaluate the reliability of Pixel Watch VO_2_ max estimates.

For heart rate, we found sufficient accuracy to quantify exercise intensity in most instances. However, we also identified measurement periods with large error among two participants. Of all metrics, the largest overall mean absolute percentage error was for energy expenditure; on a per-participant level, it ranged between 2% and 49%.

Our findings largely align with prior validation of wearables from other manufacturers. A meta-analysis of VO_2_ max estimates from Garmin, Polar and Fitbit devices reported a small mean bias and wide limits of agreement (−9.92 to 9.74 mL/kg/min) [17]. In comparison to Apple Watch VO_2_ max estimates, we found stronger agreement, with smaller bias and narrower limits of agreement [13,14,26]. In contrast, we found wider limits of agreement for heart rate than those reported for Fitbit, Apple Watch and Garmin devices [32–34]. Large and inconsistent error margins have been reported for energy expenditure across numerous manufacturers [33,35].

Many factors affect measurement accuracy, including individual physiology, environmental conditions, and device software and hardware. VO_2_ max estimation requires extrapolation of an individual’s heart rate response to exercise. However, extrapolation is challenging due to the large individual variation in heart rate response, particularly to light intensity exercise [36]. Moreover, when environmental conditions – such as terrain, gradient, or heat – increase exercise workload, the Pixel Watch 3 may misinterpret the user’s physiological response and inaccurately classify an elevated heart rate as a sign of lower VO_2_ max, rather than a reflection of increased effort. Intake of caffeine, alcohol, or medications that affect heart rate also influences VO_2_ max estimates [17,37]. Heart rate accuracy is substantially influenced by motion, which causes artefacts in the PPG waveforms used to derive measurements [16,38]. Skin contact pressure, moisture and blood perfusion also negatively impact PPG waveforms and hamper accuracy [16].

Another factor that affects accuracy is the amount of inference required to derive each metric. Many inputs are required to estimate VO_2_ max and energy expenditure: PPG waveforms, demographic information, several motion sensor measurements and GPS-derived metrics, including speed, distance, and elevation. Error from these individual inputs can compound when they are combined to generate estimates, resulting in inaccuracy [39]. In contrast, heart rate is obtained directly using PPG, reducing the amount of inference required. Because these factors vary with exercise type and environmental conditions, our results should be interpreted within the context of our treadmill protocol.

Measurement accuracy should be evaluated based on its intended use case. For clinical use, thresholds corresponding to clinically significant change may be used to determine whether accuracy is adequate. For example, a 287 kcal/day increase in free-living energy expenditure has been associated with a 32% decrease in all-cause mortality risk [10]. Accuracy that permits identification of such changes may be sufficient for certain clinical applications. For personal health and fitness monitoring, wider margins of error may suffice to provide informative approximations. In large-scale epidemiological trials, where individual error is attenuated at the population level, such measurements may provide researchers with insight into associations and risk stratification across groups.

This study had a number of strengths. Our statistical and procedural methodology, guided by the INTERLIVE consortium’s expert statements, was robust. Three clinically important metrics were compared against validated criterion methods, and heart rate analysis accounted for repeated measures to appropriately estimate variance. In addition, stringent criteria were used to confirm true criterion VO_2_ max and participants generated VO_2_ max estimates in a free-living environment, reflecting real device use. This enhances the ecological validity of our findings. Notably, this is the first study to assess the validity of measurements from the Pixel Watch 3.

Our study had several limitations. First, our sample predominantly comprised young, healthy individuals with high cardiorespiratory fitness. Only four participants were in the 50^th^ percentile of cardiorespiratory fitness or lower, whereas 14 participants were in the 90^th^ percentile. This limits the generalisability of our findings to those with low cardiorespiratory fitness, older adults and patient cohorts in particular. Second, the size and composition of our sample precluded subgroup analyses by cardiorespiratory fitness level and the investigation of physiological and environmental confounders that may have affected accuracy. We were also unable to investigate the impact of Fitzpatrick skin tone as our sample included few individuals with dark skin tones. Third, the proprietary nature of Google’s measurement algorithms limited interpretability, as well as the influence of each confounder on the final measurement. Fourth, we were unable to assess the reliability of measurements due to the study’s cross-sectional design. Given that conventional VO_2_ estimates are valuable due to their reliability rather than their validity [19], evaluation of reliability would have substantially enhanced the practical implications of our findings. And fifth, our results for heart rate and energy expenditure best reflect our laboratory-based running protocol and may have differed in alternate measurement conditions.

Recurrent validation is needed to keep pace with iterative hardware and software updates, particularly given advances in machine learning approaches for measurement algorithms. Future studies should include older adults and individuals with low cardiorespiratory fitness and should investigate contextual and physiological factors that may influence accuracy. Rigorous validation will also support transparent and explainable digital biomarkers that distil data into interpretable indicators of health for clinicians. Assessing the reliability of VO_2_ max estimates will be critical to determining the clinical applicability of longitudinal trends. Lastly, larger and more diverse training datasets should be used to develop measurement algorithms that account more adeptly for physiological differences between individuals, improving accuracy.

Our study assessed the validity of VO_2_ max, heart rate and energy expenditure estimates from the Google Pixel Watch 3. We found substantial inter-individual variability in error for VO_2_ max and energy expenditure. There was strong agreement for heart rate, despite moderate measurement variability. While the clinical use of individual VO_2_ max estimates from the Google Pixel Watch 3 is limited, the device may provide a scalable method of cardiorespiratory fitness assessment for research trials, and validation of measurement reliability is warranted to evaluate its use as an alternative to conventional submaximal VO_2_ max estimation methods. Our findings illustrate that wearable metrics require condition- and population-specific validation prior to use in public health practice.

## Supporting information

S1 File Supplemental results. Additional demographic characteristics tables, heart rate line plots and error distribution figures.
(DOCX)

S2 File Pixel Watch graphical abstract. (PNG)
