# Supplementary material for: The accuracy of VO2 max, heart rate and energy expenditure measurements from Google Pixel Watch 3
Source: PLoS One. 2026 Sep 15;21(9):e0356808. doi: 10.1371/journal.pone.0356808 (PMC13577386; doi:10.1371/journal.pone.0356808)
Supplement: Supplementary file 1 [file pone.0356808.s001.docx]

**Accuracy of VO_2_ max, heart rate and energy expenditure measurements from Google Pixel Watch 3.**

**Supplemental Appendix**

**Table of contents**

S1: Demographic characteristics of included participants for each biometric	pp. 1–2

S2: Line plots of heart rate: Polar H10 vs Google Pixel Watch 3	pp. 3–4

S3: Bar charts of absolute percentage error by participant (VO_2_ max, EE)	p. 5

S4: Box plots of absolute error distribution for VO_2_ max and EE	pp. 6–7

**Supplemental Appendix 1**

**Table S1.** Demographic characteristics of participants included in VO_2_ max analysis.

| **Characteristic** | **Value** |
| --- | --- |
| Total participants, no. | 26 |
| Female, no. (%) | 10 (38%) |
| Age, mean (SD), years | 29.12 (8.38) |
| Height, mean (SD), cm | 172.57 (9.59) |
| Weight, mean (SD), kg | 71.11 (10.77) |
| BMI, mean (SD), kg/m^2^ | 23.75 (1.83) |
| Fitzpatrick skin tone ^a^ |  |
| Type I | 2 |
| Type II | 18 |
| Type III | 5 |
| Type IV | 1 |
| CRF level ^b^ |  |
| 90^th^ percentile, no. | 9 |
| 80^th^ percentile, no. | 7 |
| 70^th^ percentile, no. | 4 |
| 60^th^ percentile, no. | 3 |
| 40^th^ percentile, no. | 3 |

**BMI:** Body Mass Index

^a^ Number of participants of each skin tone according to the Fitzpatrick Scale.

^b^ Number of participants of each cardiorespiratory fitness level, classified

according to FRIEND.

**Table S2.** Demographic characteristics of participants included in energy expenditure analysis.

| **Characteristic** | **Value** |
| --- | --- |
| Total participants, no. | 30 |
| Female, no. (%) | 12 (40%) |
| Age, mean (SD), years | 29.83 (8.58) |
| Height, mean (SD), cm | 172.71 (9.21) |
| Weight, mean (SD), kg | 72.09 (10.72) |
| BMI, mean (SD), kg/m^2^ | 24.03 (1.78) |
| Fitzpatrick skin tone ^a^ |  |
| Type I | 2 |
| Type II | 19 |
| Type III | 7 |
| Type IV | 2 |
| CRF level ^b^ |  |
| 90^th^ percentile, no. | 12 |
| 80^th^ percentile, no. | 7 |
| 70^th^ percentile, no. | 4 |
| 60^th^ percentile, no. | 4 |
| 50^th^ percentile, no. | 1 |
| 40^th^ percentile, no. | 2 |

**BMI:** Body Mass Index

^a^ Number of participants of each skin tone according to the Fitzpatrick Scale.

^b^ Number of participants of each cardiorespiratory fitness level, classified

according to FRIEND.

**Table S3.** Demographic characteristics of participants included in heart rate analysis.

| **Characteristic** | **Value** |
| --- | --- |
| Total participants, no. | 33 |
| Female, no. (%) | 12 (36%) |
| Age, mean (SD), years | 29.27 (8.45) |
| Height, mean (SD), cm | 173.54 (9.22) |
| Weight, mean (SD), kg | 72.85 (10.87) |
| BMI, mean (SD), kg/m^2^ | 24.05 (1.80) |
| Fitzpatrick skin tone ^a^ |  |
| Type I | 3 |
| Type II | 20 |
| Type III | 8 |
| Type IV | 2 |
| CRF level ^b^ |  |
| 90^th^ percentile, no. | 12 |
| 80^th^ percentile, no. | 8 |
| 70^th^ percentile, no. | 3 |
| 60^th^ percentile, no. | 5 |
| 50^th^ percentile, no. | 1 |
| 40^th^ percentile, no. | 3 |

**BMI:** Body Mass Index

^a^ Number of participants of each skin tone according to the Fitzpatrick Scale.

^b^ Number of participants of each cardiorespiratory fitness level, classified

according to FRIEND.

**Supplemental Appendix 2**

**Figure S1. Participant 28 heart rate.** Line plot of Polar H10 (red) and Google Pixel Watch 3 (blue) heart rate measurements during maximal exercise testing for Participant 28, where large deviations were observed.

**Figure S2. Participant 29 heart rate.** Line plot of Polar H10 (red) and Google Pixel Watch 3 (blue) heart rate measurements during maximal exercise testing for Participant 29, where large deviations were observed.

**Figure S3. Participant 26 heart rate.** Line plot of Polar H10 (red) and Google Pixel Watch 3 (blue) heart rate measurements during maximal exercise testing for Participant 26, an example of low measurement error.

**Figure S4. Participant 13 heart rate.** Line plot of Polar H10 (red) and Google Pixel Watch 3 (blue) heart rate measurements during maximal exercise testing for Participant 13, an example of typical measurement performance, with minor error during recording.

**Supplemental Appendix 3**

**Figure S5. Participant-level absolute error.** Bar chart illustrating the absolute percentage error for each participant included in the analysis of VO_2_ max.

**Figure S6. Participant-level mean absolute percentage error.** Bar chart illustrating the absolute percentage error for each participant included in the analysis of energy expenditure.

**Supplemental Appendix 4**

**Figure S7. Distribution of VO_2_ max absolute error.** Box plot of the absolute error distribution for VO_2_ max (mL/kg/min).

**Figure S8. Distribution of energy expenditure absolute error.** Box plot of the absolute error distribution for energy expenditure (kcal).
